# Informal workers and access to healthcare: a qualitative study of facilitators and barriers to accessing healthcare for beer promoters in the Lao People’s Democratic Republic

**DOI:** 10.1186/s12939-016-0352-6

**Published:** 2016-04-18

**Authors:** Vanphanom Sychareun, Viengnakhone Vongxay, Vassana Thammavongsa, Souksamone Thongmyxay, Phouthong Phummavongsa, Jo Durham

**Affiliations:** University of Health Sciences, Faculty of Postgraduate Studies, Vientiane, Lao PDR; University of Queensland, Faculty of Medicine & Biomedical Sciences, School of Public Health, Herston, Brisbane, Australia

**Keywords:** Beer promoters, access to healthcare, Informal workers, Informal sector, Habitus, Bourdieu, Lao PDR

## Abstract

**Background:**

Informal workers often face considerable risks and vulnerabilities as a consequence of their work and employment conditions. The purpose of this study was to examine the interplay between the experience of informal work and access to health, using as an example, female beer promoters employed in the informal economy, in the Lao People’s Democratic Republic.

**Methods:**

In-depth interviews were undertaken with 24 female beer promoters working in beer shops, restaurants and entertainment venues in Vientiane City. The recruitment strategy of snowball sampling was used. Interviews explored the beer promoter’s experience of the organization of work, perceived healthcare needs, access to healthcare and insurance, and health seeking practices. The data was analysed thematically and subsequently using Bourdieu’s concepts of habitus, capital and field.

**Results:**

Most of the beer promoters included in the study were 18 years of age, single, had worked as beer promoters for more than one year and just over half were working to support their higher education. The beer promoters demonstrated a holistic view of health, also viewing good health as contributing to being beautiful – an important attribute in their work. Many reported that their work conditions, including the noisy environment, exposure to second-hand tobacco smoke, long hours on their feet and sexual harassment negatively affected their physical and mental health. Only four participants had any form of health insurance with access to healthcare constrained by individual characteristics, health system factors and the conditions of their informal employment.

**Conclusions:**

Drawing on the work of Bourdieu, the study shows how both employment and illness are linked to habitus embodied in everyday practices, access to capital and the position the female beer promoters hold in the social hierarchy in the field of employment.

## Background

With the Millennium Development Goals (MDGs) expiring at the end of 2015, world leaders have agreed to seventeen Sustainable Development Goals (SDGs) which will guide global development policy and planning up to 2030.

To advance the global health goal (Goal 3: Ensure healthy lives and promote well-being for all at all ages) further progress needs to made in ensuring universal access to affordable and appropriate healthcare [1]. Achieving this requires reaching those employed in the informal sector who are largely excluded from any protections provided by national labour laws and regulations [2–4]. The informal workforce is estimated to comprise of one-half to three-quarters of non-agricultural employment in low- and middle income countries. Their lack of formal employer-employee relationships makes them a difficult population to reach and they are often the last to be enrolled into social protection and health insurance schemes [2–4]. Irregular incomes also contribute to informal workers being reluctant to join insurance schemes, such as community based insurance, or leads to defaults on contributions [4, 5]. Yet, job insecurity and poor working conditions often leaves informal workers particularly vulnerable to catastrophic health shocks [6]. Women comprise a large proportion of the informal employment sector and are particularly vulnerable due to their employment conditions. Women often work in the lowest paying informal jobs, employment conditions often prevent them from meeting their maternal health needs and their working conditions often subject them to physical as well as emotional and sexual abuse [5]. The Lao People’s Democratic Republic (PDR) is a lower-middle income country situated in Southeast Asia and is undergoing rapid socio-economic change, including the emergence of a large, informal but uninsured, urban workforce [4, 7–9].

Female beer promoters, a relatively new phenomenon in Lao PDR, are part of the growing informal urban workforce. Their exact number is not known, but unofficial estimates suggest that there are 8,000 to 10,000 women working in the bars, beer shops and nightclubs, as beer promoters in Vientiane Capital City [10]. Beer promoters are differentiated from beer sellers and are employed by both international and national breweries to promote the sales of beer in restaurants and other establishments [10]. They are predominantly young, female and attractive and often required to wear revealing, branded uniforms of the brewery. The working conditions of the beer promoters mean that they are vulnerable to harassment and sexual abuse compounded by broader gender and social norms [10].

Using beer promoters in the informal sector as an example, the present study draws on the work of the French theorist, Pierre Bourdieu [11–14], to provide insights into the processes and experience of informal work and access to health. Using the conceptual tools of Bourdieu [11–14], we examined how the interactions between informal employment and the healthcare system and access to social, cultural and economic capital shaped health seeking practices.

Bourdieu [11] proposed three concepts that are fundamental to understanding how individuals cope with imposed social structures: habitus, field, and capital. For Bourdieu [11] social practice is an effect of actions and interactions of agents struggling in social spaces (fields) over resources at stake and the power to set out the “rules of the game” in order to survive and pursue wealth, profit, power and distinction [15]. These practices and interactions are shaped through habitus that is, a personal set of cognitive and embodied dispositions that guide how people act and the sum of a person’s economic, social, cultural and symbolic capital. Habitus develops through socialisation experiences and thus, particular dispositions more or less reflect particular social fields with people who move through similar social contexts acquiring similar habitus [11, 13]. It is also about power relations, and in particular about those power relations that exist between social classes and that appear in the complex interactions and practices embodied in a particular habitus [13, 15].

Capital is embodied in economic, cultural, social, and symbolic forms [11]. Economic capital refers to material and financial assets and other forms of income such as wages. Cultural capital describes the skills and knowledge that are considered legitimate and useful in dominant society. Social capital relates to social connections, the utility of which depend on the depth and breadth of one’s social networks, and the volume of material resources held by people in the networks [11]. The different capitals are not independent, but are closely related to one another and effective deployment of one type of capital, can lead to the acquisition of additional capital. For example, cultural capital attained through recognized academic qualifications, can be used in the employment field to increase one’s economic capital [12]. Symbolic capital relates to any form of capital that is given positive recognition by relevant actors within the field. In the informal economy, the employer has more symbolic capital than the workers, and it is the employer who determines working conditions and which workers will be formal, and which will be informal, and it is the employer who benefits from the informality of the workforce [2]. Habitus, unawareness of the rules of the game of employment and a lack of relevant capitals, means that informal workers often passively accept their employment and work conditions. Definitions of the key concepts in Bourdieu’s framework are provided in Table 1.

**Table 1:** Definitions of key concepts in Bourdieu’s theory of practice

Concept	Definition
Field	A distinct structured social space consisting of different actors and social positions. A field is a network, structure or set of relationships in which agents struggle over desirable resources, tacitly following the rules of the game.
Capitals	Resources of value in the field. ). These forms of capital can be accumulated and transferred from one arena to another.
Economic capital	The different means of production and other forms of income, such as wages.
Social capital	Resources derived from membership in social networks and maintenance of strategic relations. The utility of which depend on the depth and breadth of one’s social networks, and the volume of material resources held by people in the networks
Cultural capital	Resources derived from formal education, training and socialisation and can contribute to the owner's financial and social advantage and is expressed in for example, style of speech, dress, or physical appearance, educational credentials and knowledge).
Symbolic capital	Status and power afforded on the basis of social recognition of the value of other forms of capital the actors possess, and that legitimate existing social hierarchies.
Habitus	Habitus is created through a social process leading to patterns that are enduring and transferrable from one context to another, but which may also change over time. It is created by the interplay between structure, capitals and dispositions. Dispositions are shaped by past events and structures, and shape current practices and structures and condition very perceptions of these practices.

## Methods

### Methodology

#### Research design

Bourdieu’s empirical work mainly drew on correspondence analysis and ethnographic studies but he did not specify a particular method for data collection and analysis and quantitative, qualitative and mixed method designs have been used [16]. This was a qualitative study that used in-depth interview (IDIs) with female beer promoters. The beer promoters’ role was to promote a specific brand of beer usually in bars, restaurants and entertainment establishments. The setting was three urban districts (Chanthabury, Sisattanak and Saysettha districts) in Vientiane capital city. These districts were purposively selected based on the large number of beer shops in these districts.

#### Sampling

The female beer promoters (*N* = 24) were recruited into the study using snowball sampling. The research staff met with potential participants in advance to explain the research and address any questions that potential participants had. Potential participants were given an information sheet and verbal consent was obtained prior to conducting the in-depth interviews. In addition, we sought approval from the participants’ workplace. Inclusion criteria was: 1) aged 18 years and older; 2) living in the city and working as beer promoters for at least the three months previous to the study; 3) part of the informal workforce; 4) able to provide informed consent. The participants had to fulfil all the criteria to be eligible. In total, there were 24 participants: 12 who worked in beer shops and 12 who worked in restaurants or entertainment venues.

#### Data collection

Data was collected in face-face interviews with a trained researcher. An interview guide was used and included questions related to socio-demographic characteristic (age, sex, education, ethnicity, marital status, type of work and contract), the organization of work, perceived healthcare needs and access to healthcare and insurance, and health seeking practices. While an interview guide was used, questions were sufficiently open to allow participants to focus on the issues that were important to them, providing the interviewer the flexibility to follow up and clarify participant ideas, and adapt interviews as the study progressed [17–19]. The interviews were conducted in the workplace before starting work or any other location preferred by the participant. Each interview took approximately two hours. Participants were asked to use a pseudonym or avoid using their names. All interviews were audio-recorded using a digital recorder and then transcribed by a professional transcriptionist with all other personal identifiers (e.g. names of district, establishments etc.)

#### Data analysis

Basic statistics were used to obtain demographic data. The qualitative data were analysed manually using thematic analysis and using both inductive and deductive approaches guided by the literature [18] and using the following categories: 1) informal workers’ working conditions, 2) employment conditions, 3) health status, 4) economic factors that drove them to work as beer promoters, 5) perceived of health and illness, 6) health care seeking process, 7) the beer promoters’ access to health care system, 8) continuity of care, and 9) utilisation of alternative medicines. Coding was done manually by two research team members. Another research team member independently coded a subset of the data to check for convergence and consistency. Each coder read and re-read the transcripts several times. Analysis continued throughout the writing process [17]. Subsequently data was analysed drawing on Bourdieu’s concepts of habitus, based on attitudes to their informal work, health and the health system; capital based on an evaluation of their characteristics including gender, age, education and their attitudes to their work; and field by looking at the individuals in the social space of the beer promoters’ work and the power relations in the social space [12, 13]

#### Translation

Interviews were undertaken and transcribed into Lao and translated into English. Where questions arose from the transcripts, they were reviewed again against the recording to check the accuracy. Almost inevitably however, in the interpretation process editorial decisions were made and some nuances may have been lost in the process. Quotes used in this paper therefore are edited translations.

## Ethics

Ethical approval was obtained from the National Ethics Committee for Health Research, Ministry of Health, Lao PDR. Verbal consent was obtained from each participant prior to interviews and was approved by the Ethics Committee. The participants were assured of privacy and confidentiality. Participants were informed that their participation was voluntary and that they could withdraw from the interview any time without notice and without consequences. The interviewers explained the purpose of the study and procedures and checked participant understanding. Participants were reimbursed 50,000 kip in cash (The exchange rate at the time was USD1 = 8000 Lao kip) as a token of appreciation for their time to participate in the study. Participants were not aware of this until after the interview had concluded.

## Results

### Socio-demographic characteristics and access to economic, social and cultural capital

The majority of the female beer promoters recruited into the study were 18 years of age and had worked as beer promoters for more than one year (*n* = 17). Eleven of the respondents were from Vientiane while 13 had migrated to Vientiane Capital City from other provinces. The majority were single (*n* = 22), had completed high school (*n* = 18), 14 were in higher education at the time of the research and four worked in additional jobs in the informal sector as well as working as beer promoters. All of the participants were ethnic Lao, the dominant ethnic group. While not in the lowest economic strata, participants needed their income from their work to either support their study and associated living expenses or to take care of their family. Many of the respondents reported that they struggled balancing their commitments as beer promoters and those of being students or working elsewhere and complained of often being tired. The income from their informal work was used to help them gain cultural capital through university or to support their family and most of them reported that in this way it also helped to reduce the financial burden of their family.

The value of their social networks was not very high and consisting of family relations, ties in the close neighbourhood or at work or in their higher education institution. Two had written contracts with the company as they had worked with the company for more than a year. Participants reported that they were organized into teams of work with each team consisting of seven to eight beer promoters and a supervisor. Usually they were picked up by the beer company each evening and dropped off at pre-identified places, which changed on a regular basis. At the end of their shift they were again picked up by the beer company and dropped off at their homes, providing a level of protection late at night. Not all beer companies had a van to pick the girls up and take them to work regardless of the location of their houses. Some of the girls lived far away from the company or beer shops and had to come to work on their own, often coming home late at night and in the dark. Most of the respondents reported that while at work they were generally not allowed to drink beer with their clients. Many felt that the conditions of their work negatively affected both their physical and mental health. Common complaints were back pain, stomachache, headache and tiredness and some said they had lost weight.

### Seeking and accessing healthcare

For minor illnesses such as cough, fever, headache or stomach problems, self-medication being the first line of treatment, often in consultation with family and friends. The quote below reflects a commonly reported practice:

*We need to consult with family and relatives about where to get good treatment, especially consulting with relatives who have more experience in utilising the different health services. (Beer promoter, 22 years old)*

Where self-medication did not resolve the issue, the next step was typically presenting at a small private clinic, which in Lao PDR, are typically owned and managed by sole operator medical officers who also work in the public sector. The public health system was often seen as the place of last resort when other treatment options had not worked or when the symptoms were perceived to be severe or the cost of treatment was uncertain but expected to be high or in health emergencies. The following quote helps to highlight a common sentiment, with public facilities and hospitals (which are public) only being seen as places to go when very sick:

*The services in the public hospitals are not so good and the services are slow, some staff do not speak nicely. If the patients do not know the health staff, they do not treat patients well. So, why if I am not severely ill, why would I go to the public facilities? However, the hospitals have more medicine and equipment. If we are getting severely sick, we have to go to the hospitals as we do not have any choice.*

The medical costs plus access to financial resources were mentioned as the most important determinants in the health-seeking process. Almost all of the participants worried about their financial and job security and for more than routine medical needs said that they would need to borrow money to cover the costs. Almost all (*n* =20) had no health insurance and were responsible for their own healthcare costs. Two had partial insurance coverage and two were insured through their family’s insurance. Some were reimbursed approximately 50 % of the cost of healthcare on presentation of a receipt from the hospital but this was unusual. None of the participants had access to a work-sponsored fund where they could borrow money to cover medical costs. In such cases or in the case of chronic disease, the young women in this study reported they would need to borrow money from people in their social networks.

The indirect costs of medicine and loss of income, concerns about being replaced and losing their job if they were absent, inconvenient opening hours in relation to the beer promoters’ work and other commitments, the unpredictability of informal payments, perceived poor quality of care and long waiting times were also important supply and demand-side factors that often acted as barriers to using public healthcare services. The quotes below are illustrative of some of the barriers and help to highlight the interactions between human, social and economic capital and access to healthcare:

*Mostly, I go to the drugstores or clinics because the services are better than the services in the hospital, not a long waiting time. In the hospitals, the health staff tell us to go to this place or to go to another place and it is confusing. The health staff do not welcome us and they do not speak nicely and sometimes we ask them a question, but they do not tell us or take good care of us. If we do not have money, they will not take care of us. And comparing the prices of the private clinics and the public, the prices are quite similar. And in the hospitals we have to give the tips to the health staff. So, it is better to go to clinics and the services are good and close to my house. (Beer Promoter, 19 years)*

*The health staff at the public hospitals are not so good as they are tired and there are many clients at the public hospitals. Sometimes, they get angry if we keep asking questions and they do not speak nicely with the patients. Sometimes, I think that they are tired with the examination of the patients, so they are not in so good a mood. Sometimes, I have to wait for long time, for example, I was in the queue, and then, other patients jumped in front of me. If we do not know anybody, we have to wait for a long time. (Beer Promoter, 19 years)*

*In the hospital, the billing system is complex because the billing system is not performed at one place. For example, I went to hospital A. I had to bring the registration book to examine in one room, then to the finance section. After that, I went to be examined in the room upstairs, after that I went to the finance office or cashier. Firstly, I did not know that I have to be examined upstairs, so I waited in downstairs until someone told me that the examination room was upstairs and I had not seen it. I think patients get easily confused for their first time to the hospital. (Beer Promoter, 19 years)*

Even with education, most of the participants did not know about community based health insurance, were not confident in navigating the healthcare system and as young women, felt unable to ask questions or assert themselves in the health setting encounter. Typically, they reported that in the medical encounter they did not reveal their position as beer promoters because they felt that this would create stigmatising attitudes from the healthcare staff, and in particular, that they would be perceived as sex workers.

The lack of knowledge related to navigating the health system as well as work-based struggles of maintaining their beer promotion duties induced feelings of anxiety, which contributed to self-medication as the first line of treatment. To overcome feelings of anxiety and uncertainty, common tactics deployed by participants were to rely on their own previous experience and the experience and knowledge in their social networks. Social networks were the first point of information on what the problem might be, which pharmacist or healthcare provider to consult, which treatment to take and to find out if anyone had contacts in any of the public health facilities that could help guide them through the systems. Where they did feel the need to use a public facility, it was usually where someone in their social network knew someone in a particular facility, albeit someone with limited seniority. While most respondents felt they could afford the medicine they purchased from the drugstores for routine, non-severe health issues, many worried about the cost of on-going care. Work condition barriers included lack of work-based insurance, concerns about taking time off, anxiety about maintaining their employment and no worker organizations to advocate for the rights of informal workers. The quotes below are illustrative and help to highlight the interactions between social and economic capital and how social networks can potentially be used to raise funds:

*“If I became very sick, I could not afford to pay. If I am admitted to the hospital, my parents will pay, however, if my parents did not have the resources, they would have to borrow from relatives and friends. If I am severely ill and a long-term illness, we have to borrow from relatives, sometimes, we have to deposit some precious things or valuable things with lenders such as car, house or land. After recovery, we will find some money and repay the lenders. I could not borrow from the Beer Company, and we do not get any insurance from them.” (BP, 19 years)*

*If I am getting so sick that I have to be admitted to the hospital for long time, there will be a high cost of hospital admission, high cost of medicines, and I do not have the money to afford to pay. The company does not take care of the health expenses and I would have to ask my family or friends (Beer promoter, aged 22 years)*

### Work and employment conditions and the symbolic power of economic capital

The brewery management, the management and clients of the restaurants and other establishments where the beer promoters work, have more economic and symbolic power than the young, female beer promoters. The brewery management set the hours of the beer promoters’ work and their conditions. Some worked for up to eight hours while others worked less. With an inconsistent income, they relied on tips from customers. Most the respondents said that were allowed to take up to three days unpaid leave if needed but if they were absent for more than this, they would probably lose their position, and have double their daily wage subtracted from their pay for the days in which they were absent (i.e. if they were absent for 5 days they would lose 10 days’ pay). Some of the beer promoters said that they were often required to undertake non-paid work for the owner or manager of the establishment where they were deployed. This included for example, cleaning or waiting at tables. While not part of their agreement, the beer promoters did not consider refusing to do this additional, unpaid work was an option.

All of the respondents reported working in poor working conditions including exposure to loud and constant noise, bright lights, second-hand smoke, and tiredness from lack breaks, standing in high heeled shoes for long periods of time and eating at irregular times, as the following illustrate:

*In our work we are often exposed to [tobacco] smoke, noisy and we stand for long hours, eat at the wrong time and sleep late. (Beer promoter, 21 years)*

*“I have to stand out for long time and I have some leg pain and pain in my feet as I wearer high shoes. (Beer promoter, aged 19 years)*

Most of the beer promoters said that they were not allowed drink beer with clients, and while some of the clients might be rude or inappropriate, they were expected to avoid any conflict with the clients. Sometimes as more beer was consumed the behaviour of the clients was reported to become increasingly inappropriate including harassment which varied from verbal abuse (from male and female clients) to unwanted touching and sexual propositions. Many felt that the female guests often looked down on them, assuming they were promiscuous and engaged in commercial sex. The beer promoters reported tolerating these behaviours and always being polite to the venue’s customers. All of the beer promoters interviewed said that their role was to serve beer to the venues customers but they were not allowed to sit or drink with clients, were expected to avoid the conflicts with clients and they had sometimes experienced difficulties with clients. The quotes below provide examples of how the unequal structures of power were manifested and how the beer promoters try to manage this:

*In terms of client, some of them look at me with not good feeling, especially the female clients, because they may think that we are working like a prostitute. But I don’t care about their perceptions; I just smile at them because I or we know ourselves, that as beer promoters, we have our honor, no matter what they think about us. I can move to another table if I feel not good with a client. Sometimes after work, I wait for the company car to take me home, some men call me and ask me to go with them at night, and I answer ‘No, thanks’. If they still call, I don’t care, I keep a little smile and not look at them. Sometimes, men say that “Girl, I can take you out of here but not to your home, OK? Come on, come with me, girl”. Mostly we all do the same - no argument. (Beer promoter, 19 years)*

*A man touched my bottom, I kept quiet with angriness inside, and he spoke out very loud among his friends and many clients like this: “girl, I am sorry I touched your bottom”. Everybody heard that and I was so shy. And I suddenly answered out loud like this: “That is fine brother, but I really don’t like it. If you really want to do that, please be a man and ask nicely and I may allow you perhaps; otherwise, I or other people may think that you have no manners or have never been to school”. Then, his friend apologized to me instead of him because he was drunk. I also added more “That is OK to drink and get drunk, but please stay aware”. Doing like that is really wrong. I am just a girl, but he is a lot older than me and he has work, I don’t want to say much more than this”. That was the moment I was really angry, but felt good to let them know. As I said, I try to talk nicely with them, as to keep a good image of the company who hire me. (Beer promoter, 20 years)*

*Sometimes, as the male guests try to touch me on my hands; if it is by accident it is OK, but if they intend to touch me, either my hand or my waist, then I must try and avoid that table. I have to avoid an argument. Some women don’t like it when I serve and their boyfriends look at me; some women even say “That’s OK, leave the bottles here, I can serve my boyfriend, and I will call you when the beer is running out. You can go to serve any other table now”. I think I know what they feel, but I am not sure they know how I feel if they say like that. But it is normal to me now. I just think that I am good-looking. Sometimes men ask me to go with them, but I don’t go and I don’t care. I have the company car to take me home and it is safe. (Beer promoter, 19 years)*

Given the beer promoters were economically dependent on their work as beer promoters, they rarely complained about their work conditions. Their informal status and anxiety about losing their job, further weakened possibilities of challenging the status quo, far removed as they were, from the aegis of formal regulations governing standards of work that protected those at the higher end of the production chain. In this way, while at work, the beer promoters experience non-respectful and sometimes even harmful treatment, that further contributed to reproducing social inequities. While the beer promoters acknowledged the detrimental effect their work conditions had on their health, their gender, class, age, position in the social hierarchy in the field of work and their life experience, shaped their habitus and understandings of what was reasonable or possible, and respondents did not overtly try to challenge the conditions of their employment or the appropriateness of behaviour of some of the clients.

### Habitus and social networks

The passive attitudes and acceptance of health system and work conditions that prevent them from seeking appropriate healthcare are not only because of anxiety and uncertainty but also their habitus. As young, single women, struggling to make ends meet and juggling varying demands, their experiences and those of other in their social networks who are from similar social strata, experience discrimination by employers, negative attitudes from healthcare workers and sustained neglect by the government which has shaped their expectations of work and healthcare.

The help of people within their social networks was sought to mobilise, when required, the required finances for healthcare, to get information about which providers to go to for which ailment, and to accompany them to health facilities to help them navigate the system, as shown below:

*Last year I had a fever, sore throat, difficult swallowing and I went to the close private clinic with my parents and my mother asked advise from our neighbors about which private clinic they suggested to go to a private clinic of a doctor working at the private clinic and at the big hospital. My mother also knew that doctor. (Beer promoter, 19 years)*

The same social networks were used to identify employment opportunities and find out which were the best beer companies to work for. Almost all of the participants in the study for example, were introduced to the work of beer promotion through people in their social network:

*I have friends working with the Beer Company, so they persuaded me to work as a beer promoter (Beer promoter, 21 years)*

*I wanted to earn some money myself and reduce my family’s expenditure, so I consulted my sister then my sister took me to apply at the company because my sister knew a beer promoter there and that they could do this job without any problem (Beer promoter, 19 years)*

The members of this network however, usually share the same habitus based on similar experiences of both the healthcare systems and informal work. Furthermore, while participants reported that these social networks were very helpful in securing work and introducing them to people who could help guide through the process of applying for work or seeking healthcare, there is a risk that dependent relationships of power and patronage develop.

## Discussion

Most of the participants in this study were young women from modest backgrounds. For the study participants their health was important and a source of capital for everyday life, enabling them to be productive. Importantly, good health was seen a contributing to beauty, an important factor in their work. Respondents reported frequent but low-risk illness episodes rather than chronic or severe episodes of poor health.

All of the young women interviewed financed their healthcare through their sometimes precarious wages, most were not enrolled in community based health insurance and all expressed concern in terms of chronic health care needs and continuity of care. A study in Vietnam of uninsured populations reported similar findings [20]. This lack of community-based health insurance is also reflective of relatively low levels of cultural capital in relation to literacy about health insurance related and economic capital.

The beer promoters’ health seeking practices were influenced and are similar to those described by beer promoters elsewhere [10]. health services supply and demand-side barriers. These health service supply and demand-side barriers can be mapped onto the domains of access, acceptability, affordability, appropriateness and availability and illustrate the interface between users and the characteristics of suppliers [10, 21–27]. These barriers have also been well described in the Lao PDR, especially for informal sector workers [9, 21, 28]. Access to healthcare and health seeking practices was also influenced by participants’ work and employment conditions and discussed within their social networks.

Inequities in access to appropriate healthcare can also be explained by interactions and hidden struggles over symbolic power in the fields of health and employment which contribute to reproducing social inequities [29]. A lack of confidence in navigating the complexities of the health system alongside poor working conditions and their lack of relevant capitals in the employment field, forced the beer promoters to passively accept both their employment conditions and uncertain healthcare and is corroborated by another study in Lao PDR, Cambodia and Vietnam [10]. Even where their conditions were detrimental to their health or not within their terms of employment, the beer promoters did not question these conditions or challenge the “rules of the game”. In contexts such as Lao PDR, these norms of passivity and modes of interacting with the health field are often compounded by power hierarchies between patients and healthcare workers and reinforced by broader societal inequities.

The apparently passive attitudes of the beer promoters are shaped by their habitus and their social network who usually share the same habitus [11, 29]. These social networks were to large degree homogenous, with limited symbolic capital and thus did not link them into social networks that would provide them with experiences and opportunities outside of their social class. These networks however were crucial resources in for the beer promoters in both finding work as a beer promoters and seeking healthcare. It was within the spaces of these networks, that the beer promoters shared practical information and advice on both employment opportunities and therapeutic options. In this way, both employment and illness were placed in their everyday social life - their acceptance of their work conditions and their health seeking was shaped by broader institutional structures and long-term historical trajectories of change and was enacted beneath the level of conscious reflection [30]. In this way, while to a large extent ambivalent about the nature of their informal status, the women in this study did not see themselves as victims. Rather, they were exercising agency and working to achieve their longer-term aspirations of tertiary level education and reduce the burden on their family. Their choices were framed by their circumstances and by what seemed reasonable to them, which in turn, was shaped by their habitus, the social structure of the labour market and their limited expectations of the role of employers in providing a safe workplace and social protection [11, 30, 31].

As with all studies, this research has some limitations. As an exploratory study, the research relied on qualitative methods only and interviews were only conducted in the capital city of Vientiane. Quantitative survey data combined with the qualitative data could have provided more primary data and allowed a more refined analysis. As a consequence this study does not claim to be representative of all beer promotion workers in the Lao PDR. In addition, time constraints on the research meant that beer promoters themselves were not engaged as researchers. Such engagement would not only have enhanced the research but may have contributed to changing the status quo and building the capacity of the beer promoters to advocate form their rights.

## Conclusion

In this paper, we have suggested that Bourdieu’s concepts of field, capital, and habitus can provide insights into the processes and experience of informal work and access to healthcare.

The study has shown a need to move away from understanding inequality in access to healthcare only through frameworks that focus supply and demand-side factors and ones that focus on individual ‘choices’ and behaviours to one that embraces broader frameworks. While the conceptual work of Bourdieu has been applied in research on inequities, few studies have used Bourdieu’s concepts in research on inequities in health-care access in low and lower-middle income countries. This paper shows that the subtle and sometimes ingrained reproduction of inequities by linking agency is linked through habitus with the structure, via field and capitals.

Drawing on Bourdieu, this study suggests that the processes that limit the access of female beer promoters to appropriate healthcare services will not be achieved by improving access to financial assets, for example through work and health system mechanisms such as insurance will not be sufficient on their own. While better access to economic capital will help, interventions also need to focus on social and institutional context and to challenge the status quo. While the informal sector is an important contributor to economic growth in countries such as the Lao PDR, it should not worsen the distribution of costs and benefits, including health outcomes and generate further inequality. Further work is required to understand what incentives would encourage the employers of informal workers to provide staff with basic protection mechanisms through contracts, minimum number of agreed hours, breaks, smoke-free workplaces and access to adequate healthcare. Further work is also required to better understand the causal mechanisms that produce and reproduce inequalities and the complex interactions of the social determinants, agency and power within the social institutions. Finally, while the challenges of reversing inequalities in access to healthcare are similar to those of other disenfranchised populations, young women, such as the beer promoters in this study, are often not a priority and their needs can easily be overlooked [32]. Yet failing to progress the well-being of young, informal workers is likely in the long-term to be detrimental to the economic well-being of nations.
